# Pre-selection of fibroblast subsets prompts prevascularization of tissue engineered skin analogues†

**DOI:** 10.1039/d2bm02022j

**Published:** 2023-05-31

**Authors:** Helena R. Moreira, Mariana T. Cerqueira, Lucília P. da Silva, Joana Pires, Mariana Jarnalo, Ricardo Horta, Rui L. Reis, Alexandra P. Marques

**Affiliations:** a 3B's Research Group, I3Bs - Research Institute on Biomaterials, Biodegradables and Biomimetics, University of Minho, Headquarters of the European Institute of Excellence on Tissue Engineering and Regenerative Medicine Portugal apmarques@i3bs.uminho.pt; b ICVS/3B's - PT Government Associate Laboratory Braga/Guimarães 4805-017 Portugal; c Department of Plastic and Reconstructive Surgery, and Burn Unity, Centro Hospitalar de São João Porto Portugal; d Faculty of Medicine - University of Porto Portugal

## Abstract

The papillary and reticular dermis harbors phenotypically distinct fibroblasts, whose functions such as maintenance of skin's microvasculature are also distinct. Thus, we hypothesized that pre-selection of the subpopulations of fibroblasts would benefit the generation of skin tissue engineered (TE) constructs, promoting their prevascularization *in vitro*. We first isolated papillary and reticular fibroblasts using fluorescence-activated cell sorting and studied the effect of their secretome and extracellular matrix (ECM) on human dermal microvascular endothelial cell (hDMEC) organization. Subsequently, we developed a bilayered 3D polymeric structure with distinct layer-associated features to house the subpopulations of fibroblasts, to generate a skin analogue. Both papillary and reticular fibroblasts were able to stimulate capillary-like network formation in a Matrigel assay. However, the secretome of the two subpopulations was substantially different, being enriched in VEGF, IGF-1, and Angio-1 in the case of papillary fibroblasts and in HGF and FGF-2 for the reticular subset. In addition, the fibroblast subpopulations deposited varied levels of ECM proteins, more collagen I and laminin was produced by the reticular subset, but these differences did not impact hDMEC organization. Vessel-like structures with lumens were observed earlier in the 3D skin analogue prepared with the sorted fibroblasts, although ECM deposition was not affected by the cell's pre-selection. Moreover, a more differentiated epidermal layer was obtained in the skin analogue formed by the sorted fibroblasts, confirming that its whole structure was not affected. Overall, we provide evidence that pre-selection of papillary and reticular fibroblasts is relevant for promoting the *in vitro* prevascularization of skin TE constructs.

## Abstract 2

Pre-selection of papillary and reticular fibroblasts is relevant in promoting the *in vitro* pre-vascularization of skin TE constructs and offers advantages in terms of representation of tissue functionality being therefore a valuable study platform.

## Graphical abstract

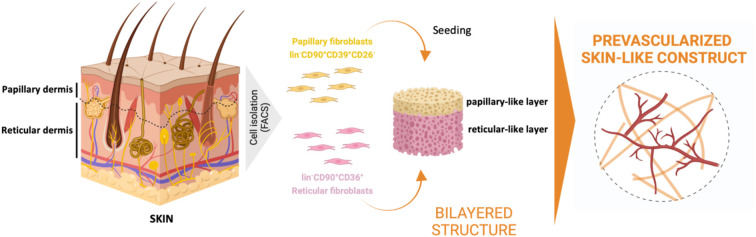

## Introduction

The skin dermis plays a crucial role in establishing microenvironments that regulate tissue physiology. This connective tissue is divided into the upper papillary and the lower reticular layers, which differ both structurally and functionally.^1^ The papillary dermis is a loose matrix containing fibroblasts at a high density and microvascular and sensory components.^2^ While the microvascular component provides nutrients to the epidermis,^3^ contributes to the skin's immune surveillance^4^ and helps in regulating the temperature of the skin and thus the body as a whole,^5^ the sensory components allow nociception and perceiving temperature and touch.^6^ In turn, the reticular dermis comprises a lower number of cells in a denser matrix involving the skin appendages (sebaceous and sweat glands and hair follicles), in addition to other sensory components.^2^ This matrix besides providing strength and elasticity to the skin allows the detection of hair movement, skin deflection, pressure, and vibration through the neural receptors.^6^ While much is still to be understood, these differences have also been linked to the distinct functional identities of fibroblasts found in the papillary and reticular dermis.^7^

The distinct identities of dermal papillary and reticular fibroblasts have long been alleged,^8^ however it was only some years ago that they were confirmed in mouse skin.^7^ Along development, fibroblasts in the mouse dermis switch from a homogeneous population—Dlk1^+^Pdgfr-α^+^Lrig1^+^ at embryonic day (E) 12.5—to two subpopulations at E16.5—Dlk1^+^Pdgfr-α^+^ cells in the reticular dermis and Lrig1^+^CD26^+^Pdgfr-α^+^Dlk1^−^ cells in the papillary dermis. From E16.5, the developing dermis undergoes fate restrictions such that cells in the upper dermis that express Lrig1 give rise to papillary fibroblasts, whereas Dlk1^+^ cells in the lower dermis give rise to the reticular ones.^7^ Because many of the cell surface markers identified in the developing mouse dermis were not conserved in humans,^9^ distinguishing human fibroblast subpopulations is still uncertain. Data generated from isolated superficial and lower dermal layers are also ambiguous since fibroblast identity is not spatially restricted within the dermis, but instead defines an opposing gradient of papillary and reticular fibroblasts from the skin surface to the deep dermis.^9,10^ It was only recently that two major human dermal fibroblast subpopulations were identified using a combination of spatial and single-cell transcriptional profiling.^9^ This work identified the lin^−^CD90^+^CD36^+^ population, which expresses high levels of ACTA2, MGP, PPARγ, and transglutaminase-2 (TGM2) in the lower dermis, and the lin^−^CD90^+^CD39^+^CD26^−^ subset, which is characterized by the expression of specific collagen chains, such as COL6A5, in the upper dermis. However, there is still no agreement regarding the specific signature of papillary fibroblasts, as they have also been identified as lin^−^CD90^−^FAP^+^ cells expressing high levels of CD26,^10^ podoplanin (PDPN), and netrin-1.^10,11^

Despite the uncertainty regarding the markers that characterize fibroblast subpopulations, *in vitro* studies have confirmed that papillary and reticular fibroblasts, isolated from dermatomed skin tissue at different depths, have distinct features. Papillary fibroblasts showed higher *in vitro* proliferation rates and longer replicative lifespans than their reticular counterparts.^8,12^ Additionally, these are more sensitive to the culture density, slowing their exponential growth if at lower densities.^8,12–14^ Several other studies have also suggested that fibroblast matrisome and secretome appear to be dictated by their location within the skin dermis and also by specific interactions with neighbouring cells. Distinctively, the expression of collagen type VII (COLVII) is upregulated in papillary fibroblasts when compared to the reticular subset,^15,16^ which is consistent with the role of papillary fibroblasts in promoting the dermo–epidermal junction.^17^ Moreover, type IV collagen was evenly expressed in the presence of papillary fibroblasts forming an uninterrupted layer resembling the basement membrane in which laminin 5 and fibronectin were also found.^18^ Interestingly, papillary fibroblasts released smaller amounts of keratinocyte growth factor (KGF) than the reticular ones^14,18^ but the exact implications of this in the communication with keratinocytes were not established. Skin dermis architecture also seems to determine the interactions between fibroblasts and other cell types such as vascular ones. Indeed, papillary fibroblasts supported the formation of vessel-like structures by macrovascular endothelial cells which has been not only related to secreted factors such as vascular endothelial growth factor (VEGF) or hepatocyte growth factor (HGF),^19,20^ but, importantly, also to the deposited extracellular matrix (ECM).^21^ Components such as collagen types I and III have been suggested to be deposited in higher amounts by papillary fibroblasts^22^ while others have alluded to the presence of proteins other than collagen I and elastin in the surrounding of the capillary loops in the papillary dermis.^23^ Additionally, MMP-1 is highly secreted by papillary fibroblasts^21^ which, although still to be proven, can be related to the endothelial cells sprouting through the degraded ECM during angiogenesis.

Taken together, all these works suggest that fibroblast subpopulations generate and modulate their own and neighbouring microenvironments, which might be critical in the construction of skin tissue-engineered (TE) constructs that better reflect the native tissue. To the best of our knowledge, so far only two approaches have considered the use of both subpopulations combining layered cell-laden collagen scaffolds.^24,25^ Yet, the generation of a dermal-epidermal skin equivalent did not follow, and the communication between fibroblast subpopulations and neighbouring cells in this context has not been addressed. Thus, herein, we hypothesized that pre-selection of the two subpopulations of fibroblasts would promote *in vitro* prevascularization of skin TE constructs. We took advantage of papillary and reticular fibroblasts isolated using fluorescence-activated cell sorting (FACS) based on recently revealed markers^9^ and studied their effect on endothelial cell organization, considering both secretome and ECM production. We then developed a bilayered 3D polymeric structure with distinct layer-associated features capable of housing the sub-populations of fibroblasts, generating an *in vitro* vascularized skin bilayer 3D structure.

## Materials and methods

### Human keratinocytes and endothelial cell isolation and culture

All experiments were performed in accordance with the Declaration of Helsinki. Human skin samples were collected after written informed consent under collaboration protocols approved by the Ethical Committee of Hospital S. João, Porto, Portugal (Nr 477/2020) and the Ethical Committee for Research in Life and Health Sciences of the University of Minho (CEICVS 135/2020). Cells were isolated from the skin specimens of healthy donors (IMC 20.8–26.8) undergoing abdominoplasties. Skin pieces were cut into small fragments and incubated with 2.4 U mL^−1^ of dispase (BD Biosciences, USA) overnight at 4 °C. The epidermis was peeled off and digested at 37 °C for 8 min using 0.05% (w/v) trypsin/ethylenediaminetetraacetic acid (EDTA, Invitrogen, UK). Digestion was stopped with fetal bovine serum (FBS, Invitrogen, USA) and epidermal cells were scraped from the remaining matrix. The cell suspension was filtered through a sterile 100 μm cell strainer, centrifuged (300*g*, 5 min), re-suspended and cultured in the keratinocyte serum free media (Life Technology, Scotland) supplemented with human recombinant epidermal growth factor (5 ng mL^−1^, Gibco, USA), bovine pituitary extract (50 μg mL^−1^, Gibco, USA), 1% (v/v) penicillin–streptomycin solution (Lonza, Switzerland) and the ROCK pathway inhibitor Y-27632 (10 μM, STEMCELL Technologies, Canada). Cultures were maintained at 37 °C in a humidified tissue culture incubator with a 5% CO_2_ atmosphere. hKCs were routinely passaged with TryplE™ Express (Life Technologies, UK) and re-suspended at a density of 1 × 10^5^ cells per cm^2^.

Human dermal microvascular endothelial cells (hDMECs) were obtained from the dispase solution and cultured in 0.7% (w/v) gelatin (Sigma, USA) coated flasks with EGM-2 MV (Lonza, USA). The cells were passaged at 70–90% confluence and used at passage (P) 3–4.

### Papillary and reticular fibroblast sorting and flow cytometry analysis

After the removal of the epidermis, the dermis was digested overnight at 37 °C using the whole skin dissociation kit (Miltenyi Biotec, USA) as per the manufacturer's instructions. A suspension of freshly isolated human dermal fibroblasts with 7 × 10^6^ cells per mL was incubated with the surface markers antibodies (ESI Table 1†) for 30 min at room temperature (RT). After washing in PBS, the cells were resuspended and then cultured in minimal essential medium (α-MEM, Invitrogen, USA) supplemented with 10% (v/v) FBS and 1% (v/v) PenStrep. The selection of the lin^−^ (*i.e.* CD324^−^CD45^−^CD31^−^)CD39^+^CD26^−^ population corresponding to the papillary subpopulation, and the lin^−^CD36^+^ population corresponding to the reticular subpopulation, was performed using a FACSAriaIII cell sorter and FACSDiva software (BD Biosciences, USA).

Fibroblast subpopulations at P1 and P2, were also analyzed with the same apparatus after being labelled at RT for 20 min with fluorochrome-labeled antibodies (ESI Table 2†) according to the manufacturer's recommended concentrations.

### Cell proliferation analysis

Fibroblast subpopulation cells in P1 and P2 were seeded at 2 × 10^3^ cells per cm^2^ and cultured for up to 7 days. Cell proliferation was determined using the PicoGreen dsDNA assay kit (Invitrogen, USA) following the manufacturer's instructions.

### Matrigel assay

Matrigel (Corning, USA) was added to 96 well plates that were then kept in a humidified incubator for 30 min. hDMECs were seeded on the top of the formed gel at a density of 1.3 × 10^4^ cells per well and incubated for 24 h in a humidified incubator at 37 °C, 5% of CO_2_ in the presence of filtered fibroblasts conditioned media obtained from 3 day cultures of cells seeded at 2 × 10^4^ cells per cm^2^ plus a starvation period of 24 h in α-MEM without FBS. hDMECs cultured in α-MEM and in EGM-2 MV-containing VEGF were included respectively as negative and positive controls of the assay. The organization of cells into capillary-like structures was assessed after 8 h and 24 h. Micrographs were taken using an Axio Observer inverted microscope with the ZEN Blue 2012 software (Zeiss, Germany). Formation and organization of the capillary-like structures were quantitatively analyzed using an angiogenesis plug-in^47^ for ImageJ2 (v2.3.0/1.53 m).

### Growth factor quantification

The levels of VEGF, FGF-2 (R&D Systems, UK), HGF (Millipore, USA), IGF-1 (Elabscience, USA) and Angio-1 (Booster Biological Technology, USA) in the fibroblast conditioned media were quantified by ELISA. Assays were carried out according to the manufacturer's instructions and the absorbance of each sample was read at 450 nm using a Varioskan Flash multimode plate reader. DNA values were used to normalize the results.

### Collagen and non-collagenous protein quantification

Sorted fibroblasts were seeded at a density of 5 × 10^4^ cells per cm^2^ to maximize ECM deposition. After 3 days, the cells were fixed with 10% (v/v) formalin for 24 h at RT. The production of collagen (COL) and non-collagenous (NCOL) proteins was determined using the Sirius Red/Fast Green collagen staining kit (Chondrex Inc., USA) following the manufacturer's instructions. Absorbances were measured at 540 nm (Sirius red) and 605 nm (fast green) using the Varioskan Flash multimode plate reader. The color equivalences were used to calculate the quantity of COL and NCOL proteins.

#### Fibroblast and hDMEC co-cultures

Sorted fibroblasts were seeded at sub-confluency (5 × 10^4^ cells per cm^2^) and inactivated with mitomycin C (100 μg mL^−1^, Sigma, USA) for 4 h at RT after 3 days of culture. hDMECs were seeded on the top at a density of 2.5 × 10^4^ cells per cm^2^ and co-cultures were kept for 7 days in the EGM-MV VEGF medium.

### Immunocytochemistry

Cells were fixed with 10% (v/v) formalin for 24 h at RT, permeabilized with 0.2% (v/v) Triton X-100 (Sigma-Aldrich, USA) for 30 min at RT and unspecific staining was blocked with 3% (w/v) bovine serum albumin (BSA, Sigma-Aldrich, USA) for 1 h. Afterwards, the cells were incubated overnight at 4 °C with anti-human primary antibodies (ESI Table 2†) diluted in 1% (w/v) BSA solution in PBS. After washing with PBS, the samples were incubated for 1 h at RT with the secondary antibodies (ESI Table 2†) prepared in 1% (w/v) BSA solution in PBS. Nuclei were counter-stained with 4′,6-diamidino-2-phenylindole (DAPI, Biotium, USA). The cells were observed using an Axio Observer inverted microscope with the ZEN Blue 2012 software.

### Cytoskeleton staining

For the visualization of the cytoskeleton F-actin fibers and nuclei, cells were fixed with 10% (v/v) formalin for 1 h at RT, and stained with phalloidin-TRITC (Sigma, USA) and DAPI for 1 h at RT. Cells were observed using an Axio Observer inverted microscope with the ZEN Blue 2012 software.

### Bilayer gellan gum-based sponge-like hydrogel fabrication

Gellan gum (0.25% (w/v), GG, Sigma, USA) was chemically modified with vinyl moieties (DVS) as previously described.^26^ GG/GGDVS-RGD sponge-like hydrogels were prepared similarly to the GG sponge-like hydrogels^27,28^ but with modifications using a two-step approach. Briefly, a solution of 0.5% (w/v) GGDVS was reacted with thiol-cyclo-RGD (RGD, Cyclo(-RGDfC), >95% purity, GeneCust Europe) for 1 h at RT. Meanwhile, a 1% (w/v) GG solution was prepared by dissolving gelzan powder at 90 °C for 30 min. The GG solution was allowed to reach 40 °C before mixing with the GGDVS-RGD and casting into the desired mold. The hydrogel was progressively formed until room temperature was reached. Afterwards, hydrogels were frozen at −80 °C overnight and then freeze-dried (Telstar, Spain) for 24 h to obtain the reticular-like layer. The second layer was then added by pipetting the GG/GGDVS-RGD solution (75% of the volume used for the first layer) on top of the reticular-like layer. After stabilization, the constructs were frozen at −80 °C overnight and then freeze-dried (Telstar, Spain) for 24 h to obtain the bilayer structures. Cylindrical scaffold samples of 5 mm diameter were cut using a metal punch for further analysis. Bilayer sponge-like hydrogels were formed after rehydration of the dried polymeric networks.

### Scanning electron microscopy

Scanning electron microscopy (SEM) was used to analyze the microstructure of the dried polymeric networks. Prior to analysis, samples were sputter coated with a mixture of gold–palladium. A JSM-6010LV (JEOL, Akishima, Japan) microscope, operating at an accelerating voltage of 15 kV was used to capture images.

### Micro-computed tomography (μ-CT)

The sponge-like hydrogels’ microarchitecture was analyzed using a high-resolution X-ray microtomography system SkyScan 1072 scanner (SkyScan, Kontich, Belgium). Samples were scanned in the high-resolution mode using a pixel size of 11.31 μm (magnification of 23.30×) and an integration time of 1.7 s. The X-ray source was set at 35 keV of energy and 215 μA of current. Representative datasets of 150 slices were transformed into a binary picture using a dynamic threshold of 45e225 (gray values) to distinguish polymer material from pore voids. Pore size was obtained using a CT analyzer (v1.5.1.5, SkyScan).

### Compressive tests

The sponge-like hydrogels (hydrated in PBS for 24 h, at RT) were tested under static compression using an Instron 5543 (Instron, Norwood, MA, USA). Samples (13.4 mm in diameter and 3 mm in height) were subjected to a pre-load of 0.1 N and tested up to 60% strain, at a loading rate of 2 mm m^−1^. The compressive modulus was determined from the most linear part of the stress/strain curves using the secant method.

### Water uptake quantification

Dried polymeric networks were immersed in α-MEM for up to 72 h at 37 °C, to determine the water uptake profile. Samples were weighed prior to immersion (*W*_d_) and after each time point (*W*_w_) to calculate the percentage of water uptake with time (eqn (1)).

1 Water uptake (%) = (*W*_w_ − *W*_d_)/*W*_d_ × 100

### Cell-laden bilayer sponge-like hydrogels

A suspension containing 0.5 × 10^6^ reticular fibroblasts was added to the reticular-like layer of the bilayer GG sponge-like hydrogel. Structures were transferred to polycarbonate transwells with the reticular part facing down and kept in culture for 7 days in α-MEM, supplemented with 10% (v/v) FBS and 1% (v/v) antibiotic/antimycotic. Afterwards, a 2.5 × 10^6^ cell suspension containing 1 : 4 papillary fibroblasts : hDMECs was added on the top papillary-like layer. Constructs were cultured for another 7 days in EGM-2 MV-VEGF. hKCs (0.5 × 10^6^) were then seeded on the surface of the papillary-like layer and cultured for 3 days in KSFM. This medium was then replaced by FAD complete medium (110 mL DMEM (Sigma Aldrich, USA), 110 mL DMEM : Ham's F12 medium (Sigma Aldrich, USA) supplemented with 1.8 × 10^−4^ M adenine (Sigma Aldrich, USA), 10% (v/v) non-inactivated FBS, 0.5 μg μL^−1^ hydrocortisone (Sigma Aldrich, USA), 5 μg mL^−1^ insulin (Sigma Aldrich, USA), 10^−10^ M cholera toxin (Sigma Aldrich, USA), 10 ng mL^−1^ epidermal growth factor (EGF, Peprotech, UK), 1.8 mM calcium chloride (Merck, Germany) and 1% (v/v) PenStrep), and the constructs were cultured for an additional 14 days.

To evaluate the retention of the fibroblasts’ subpopulations in the bilayer construct, reticular fibroblasts were labelled with Cell Tracker™ Orange CMRA (Invitrogen, USA) and papillary fibroblasts with Cell Tracker™ Green CMFDA before seeding, following the manufacturer's instructions.

### Histological analysis

The constructs were fixed in 10% (v/v) formalin, dehydrated, embedded in paraffin (Thermo Scientific, USA) and cut into 4.5 mm sections. The sections were stained with Haematoxylin & Eosin (Sigma, USA) and Masson's Trichrome kit (Bio-Optica, Italy) following the routine protocols. For immunohistochemistry, sections were deparaffinized in xylene, re-hydrated and boiled for 5 min in Tris-EDTA buffer (10 mM Tris Base, 1 mM EDTA solution and, 0.05% (v/v) Tween 20, pH 9) for antigen retrieval. The cells were then permeabilized with 0.2% (v/v) Triton X-100 and non-specific staining was blocked with 2.5% (v/v) horse serum (Vector Labs, USA). Primary antibodies (ESI Table 3†) were incubated overnight at 4 °C. After washing with PBS, the samples were incubated for 1 h at RT with the secondary antibody (ESI Table 3†) in 1% (w/v) BSA solution in PBS. Nuclei were counter-stained with DAPI. The samples were observed using an Axio Observer inverted microscope with the ZEN Blue 2012 software.

### Image analysis

Five images of random fields were acquired for each condition and independent experiment and used to calculate the cell area with the Cell Profiler software (v 4.2.1) and to count the vessel-like structures using ImageJ software. The vessel-like structures were considered to have hollow continuous CD31^+^ cell arrangements. The number of vessel-like structures is presented as an average of the counted fields and expressed as the number of vessels mm^−2^.

### Statistical analysis

GraphPad Prism software (v 8.2.1, La Jolla, USA) was used to perform the statistical analysis. Data were analyzed using the Shapiro–Wilk normality test. A one-way or two-way analysis of variance (ANOVA) with a Tukey multiple comparison post-test was used to analyze the results with a normal distribution. Otherwise, data were analyzed with the two-tailed unpaired Mann–Whitney test or Kruskal–Wallis test with Dunn's multiple comparison post-test. Significance was set to *p < 0.05.

## Results

### Fibroblast subpopulation phenotype

**Fig. 1:**
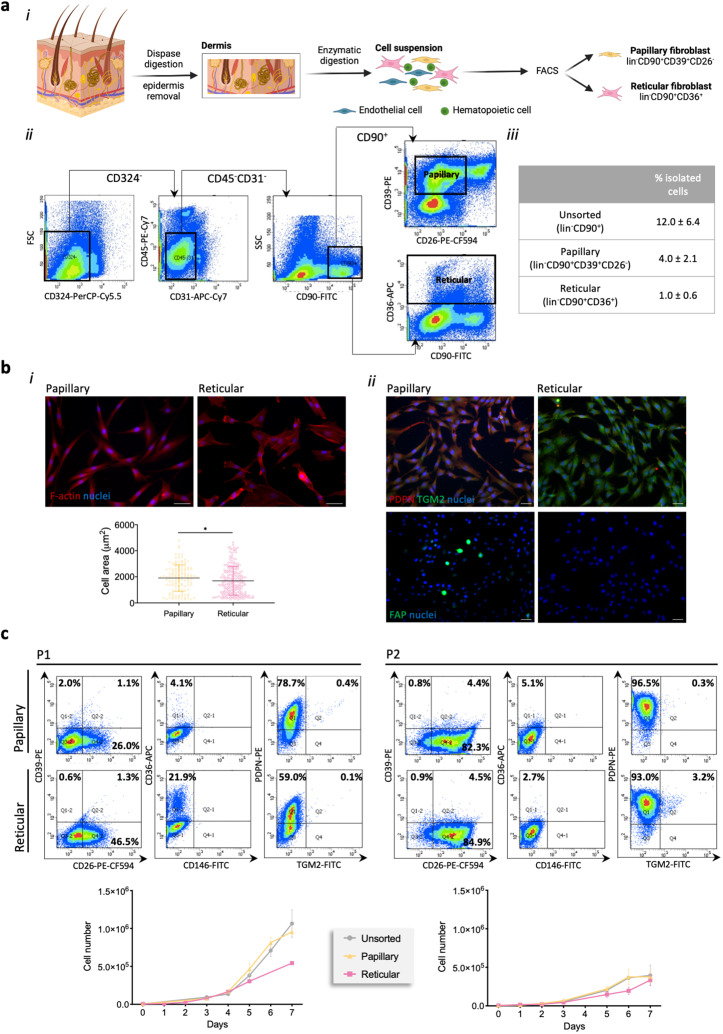
Phenotype of the fibroblast subpopulations. (a) (i) Schematics of the procedure followed for the isolation of papillary and reticular fibroblasts and (ii) the representative gating strategy of the sorting. After negative selection for CD324^−^, CD45^−^ and CD31^−^ and positive selection for CD90^+^, two populations were FACS sorted – CD39^+^CD26^−^ cells corresponded to the papillary fibroblasts, while CD36^+^ cells to the reticular fraction. (iii) Percentage of isolated fibroblasts after sorting. (b) (i) Representative images of the F-actin stained cytoskeleton and respective quantification of the area of the cells. Scale bar = 50 μm. (ii) Representative immunocytochemistry images of the expression of podoplanin (PDNP), transglutaminase-2 (TGM2) and fibroblast activated protein (FAP) after isolation. Scale bar = 100 μm. Cell nuclei were counterstained with DAPI. Individual channel images are presented in ESI Fig. 2.† (c) Phenotype of the papillary and reticular fibroblasts after passage 1 (P1) and 2 (P2) determined by flow cytometry and respective representation of their proliferation as per DNA quantification along the culture time. Quantitative results are expressed as the mean ± standard deviation where *n* = 3, * *p* < 0.05, in the two-tailed unpaired Mann–Whitney test.

The uncovering of human dermal fibroblast subpopulations has confirmed the existence of two subsets particularly localized in the upper or lower dermis.^9^ Based on these findings, we assumed these cells respectively as papillary and reticular fibroblasts and flow sorted them from the digested dermis after enzymatic removal of the epidermis (Fig. 1ai). We first started to select the cells negative for epidermal-(CD324), hematopoietic-(CD45) and endothelial-associated (CD31) markers, the lineage negative (lin^−^)(*i.e.* CD324^−^CD45^−^CD31^−^) dermal cells, and then the positive cells for CD90 within that group (Fig. 1aii). This selected sub-set of cells corresponded to 12.0 ± 6.4% (*n* = 6) of the initial population (Fig. 1aiii). From this, two distinct human fibroblast subpopulations were sorted, the lin^−^CD90^+^CD39^+^CD26^−^ subpopulation corresponding to the papillary subset and the lin^−^CD90^+^CD36^+^, the reticular subset (Fig. 1aii). Analysis of both subpopulations showed that while papillary fibroblasts correspond to 4.0 ± 2.1% of the initial population (about 35% of the lin^−^CD90^+^ population), 1.0 ± 0.6% (about 13% of the lin^−^CD90^+^ population) were reticular fibroblasts (Fig. 1aiii and ESI Fig. 1†). When put in culture (passage 0), papillary fibroblasts displayed a more spindle-shaped morphology, while a more epithelioid shape with a significantly lower surface area (*p* < 0.05) was observed for the reticular fraction (Fig. 1bi). This apparently different phenotype was further confirmed by the high expression of podoplanin (PDPN) and fibroblast activation protein (FAP) in the papillary subpopulation, and of transglutaminase-2 (TGM2) in the reticular one (Fig. 1bii and ESI Fig. 2a†). Unsorted fibroblasts expressed all the analysed markers as expected (ESI Fig. 2b†). Considering that *in vitro* culture conditions can lead to the loss of phenotype, we then assessed the expression of CD39, CD36 and CD26 as these are the differentiating markers in the lin^−^CD90^+^ population, and of CD146, a perivascular marker that has also been attributed to the reticular fraction,^10^ after passage 1 and 2. The expression of CD39 and TGM2 was almost lost in papillary fibroblasts after P1, while the percentage of cells expressing PDPN increased with the passage. Additionally, the cells initially identified as papillary fibroblasts started to express CD26 being around 82.3% of the whole cell population at P2 (Fig. 1c). Interestingly, the expression of CD36 was very low (4–5%) and did not change with the passage. Regarding the reticular fibroblasts, the expression of CD36 was only present in 21.9% of the population at P1, being almost absent at P2. Like for the papillary sub-population, the expression of TGM2 was lost upon culture and the percentage of cells expressing PDPN increased with the passage. The percentage of initially reticular fibroblasts expressing CD26 also increased with the number of passages. The expression of CD146 was absent in both subpopulations and independent of the passage. Moreover, these phenotypic changes were associated with alterations in the proliferative rate of the subpopulations which was identical at P2 and lower than that at P1. These results demonstrate that there are phenotypic changes in both cell populations after culture, therefore, in the following experiments only cells at P1 were used, it was impossible to use cells P0 due to limited numbers.

### Fibroblast influence on endothelial cell organization

**Fig. 2:**
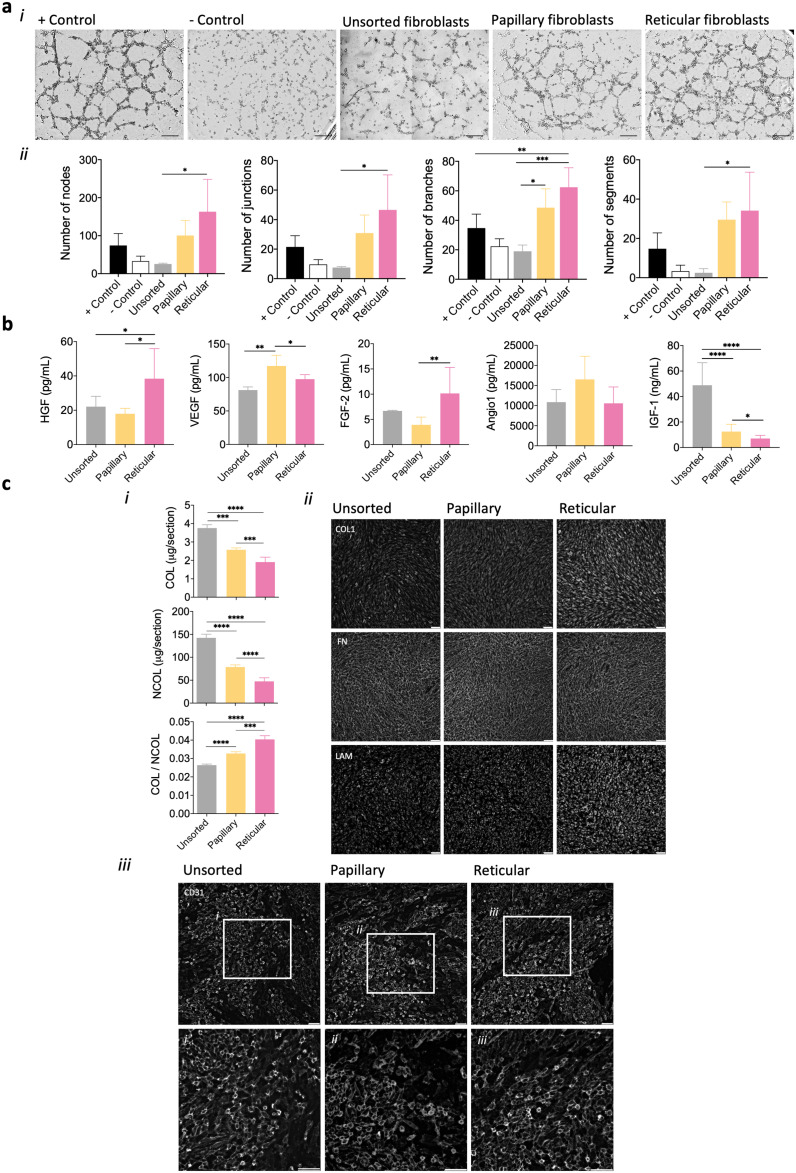
Ability of fibroblast subpopulations to signal microvascular endothelial cells. (a) (i) Tubular-like structure formation after 24 h by hDMECs cultured on matrigel with conditioned media obtained from unsorted, papillary and reticular fibroblasts, and respective (ii) number of nodes, junctions, branches and segments. Controls were set with the VEGF-containing medium (+Control) and with α-MEM (−control). Scale bar = 100 μm. (b) Amount of growth factors secreted by unsorted, papillary and reticular fibroblasts as quantified in the conditioned media. (c) (i) Amount of collagen (COL) and non-collageneous (NCOL) proteins secreted by the different subpopulations of fibroblasts and respective (ii) representative immunocytochemistry images of the expression of collagen I (COL1), fibronectin (FN) and laminin (LAM) after 3 days of culture. Scale bar = 100 μm. (iii) hDMECs (CD31^+^) organization on the inactivated fibroblasts after 7 days of culture. (i–iii) Higher magnification of the areas outlined in the upper panel. Scale bar = 100 μm, 50 μm. Quantitative results are expressed as the mean ± standard deviation where *n* = 3, *p < 0.05, **p < 0.01, ***p < 0.001, ****p < 0.0001, one-way ANOVA with Tukey multiple comparison post-test or Kruskal–Wallis test with Dunn's multiple comparison post-test.

Fibroblasts have been known to influence endothelial cell behaviour in a paracrine manner due to their angiogenic secretome.^19,20^ To understand whether the subpopulations we isolated could differently influence microvascular endothelial cells through secreted factors, a Matrigel assay with the conditioned media from their cultures was performed. A strong stimulation of capillary-like network formation was attained when media collected from papillary and reticular fibroblasts were used (Fig. 2ai). The analysis of several angiogenic parameters of the formed capillary-like network showed that the medium from the reticular fibroblasts seemed to lead to the formation of a more complex and interconnected capillary-like network (Fig. 2aii and ESI Fig. 3, ESI Fig. 4†). Significant differences (*p* < 0.05) were observed in comparison with unsorted cells (except for the number of meshes), but not with the papillary sub-population. Moreover, the time taken for the capillary-like structures to form in the presence of the fibroblast's conditioned media (24 hours) was higher than that in the positive control (8 hours) (ESI Fig. 5†). In order to better comprehend which angiogenic factors were being secreted by both papillary and reticular fibroblasts, HGF, VEGF, FGF-2, Angio-1 and IGF-1^29–32^ were quantified (Fig. 2b). The conditioned medium from papillary fibroblasts was significantly enriched (*p* < 0.05) in VEGF and IGF-1 in comparison with the one from reticular fibroblasts. In turn, the reticular fibroblast conditioned medium showed significantly higher (*p* < 0.05) HGF and FGF-2 amounts than the medium from papillary cells. Yet, the differences observed in the FGF-2 total secretion were due to the number of cells under each condition and do not reflect a higher level of secretion by reticular fibroblasts (ESI Fig. 6†). The level of all the factors in the subpopulation's media was significantly higher (*p* < 0.05) than that in the medium from the unsorted cells, except for IGF-1. Although there were no significant differences in the amount of Angio-1 among conditions, there is a tendency for higher secretion by the papillary fibroblasts (Fig. 2b), as sustained by the normalized data (ESI Fig. 6†).

In addition to the paracrine effect, it is known that fibroblasts also indirectly regulate endothelial cells’ activity through their ECM.^19^ Thus, we analyzed the ECM secreted by the subpopulations of fibroblasts and its effect on hDMEC organization. Papillary fibroblasts deposited significantly higher amounts of COL and NCOL proteins (*p* < 0.05) than reticular fibroblasts, although resulting in a significantly lower COL/NCOL ratio (*p* < 0.05) (Fig. 2ci). Moreover, it seems that the highest deposition of collagen type 1 and laminin was associated with the reticular subset (Fig. 2cii), while no substantial differences were observed in the deposited fibronectin among conditions. However, the organization of microvascular endothelial cells was not significantly affected, forming large colonies on the top of the fibroblasts independently of the subpopulation (Fig. 2ciii and ESI Fig. 7†). Unsorted fibroblast secreted significantly higher (*p* < 0.05) amounts of both COL and NCOL proteins than the subpopulations with a deposition pattern similar to the one observed for the papillary subset (Fig. 2ci and ii), however, the organization of hDMECs did not differ from what was observed for the subpopulations (Fig. 2ciii and ESI Fig. 7†).

### Bilayer spongy-like hydrogel properties

**Fig. 3:**
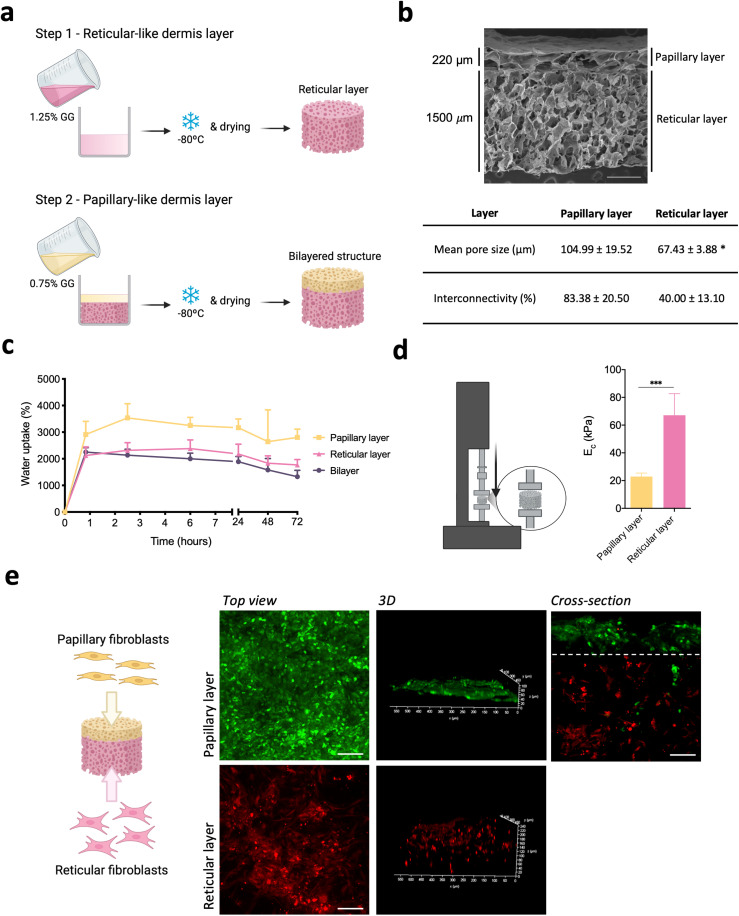
Bilayer structure preparation and characterization. (a) Schematic representation of the fabrication of the bilayer gellan gum (GG) structure. (b) Representative SEM micrograph of the bilayer dried polymeric structure and respective mean pore size and interconnectivity obtained by μ-CT analysis. Scale bar = 500 μm. (c) Water uptake ability of the bilayer structure and of the respective layers when processed independently. (d) Schematic representation of the compressive test and respective compressive modulus (*E*_c_) values of the respective layers when processed independently without cells. (e) Schematic representation of the seeding of the subpopulations of fibroblasts in the different layers of the bilayered structure and representative images of papillary (green, Cell Tracker™ Green CMFDA) and reticular fibroblasts (red, Cell Tracker™ Orange CMRA) after 3 days of culture. Scale bar = 100 μm. Quantitative results are expressed as the mean ± standard deviation where *n* = 5, *p < 0.05, ***p < 0.001, two-tailed unpaired *t*-test.

As mentioned before, papillary and reticular fibroblasts are respectively localized in the upper and lower dermis thus, we fabricated a bilayer structure integrating both the papillary- and reticular-like dermal parts to accommodate each one of those cells (Fig. 3a). The structural continuity of the bilayer structure was evident, with the individual layers being seamlessly integrated (Fig. 3b). Each layer showed a homogeneous pore structure with a high degree of pore interconnectivity. A thin 220 μm thick layer with a mean pore size of 104.99 ± 19.52 μm and 83.38 ± 20.50% pore interconnectivity outlined the papillary-like layer. The reticular-like part comprised a 1500 μm thick layer with a lower mean pore size (67.43 ± 3.88 μm) and a pore interconnectivity of 40.00 ± 13.10%.

The bilayer structures were prepared in a dry state and formed spongy-like hydrogels after hydration, as previously demonstrated for other structures processed by the same methodology.^27,28^ With the fabricated bilayer structures, an equilibrium was reached as early as 1 h after immersion in α-MEM, after taking up 2134 ± 224% of their weight in water (Fig. 3c). When comparing each layer processed independently, we found that the papillary-like layer was able to take up 3534 ± 533% of its weight in water, while 2317 ± 289% was retained by the reticular-like part. We further assessed the compressive modulus of the hydrated structure—sponge-like hydrogel—after the water uptake equilibrium was reached (Fig. 3d). The compressive modulus of the papillary-like and the reticular-like parts when analyzed separately were 22.9 ± 2.5 kPa and 67.1 ± 15.5 kPa, respectively.

Then we wanted to understand if the overall and layer-specific properties of the bilayer structure were suitable for retaining the respective fibroblast subpopulations in the intended location. More than 90% of both papillary and reticular fibroblasts remained in their destined layer, despite a minor escape of papillary fibroblasts to the reticular-like layer (Fig. 3e). Independent of the fibroblast subpopulation, cells adhered to the material homogenously, colonizing the structure layer into which they were seeded.

### *In vitro* vascularized skin-like constructs

**Fig. 4:**
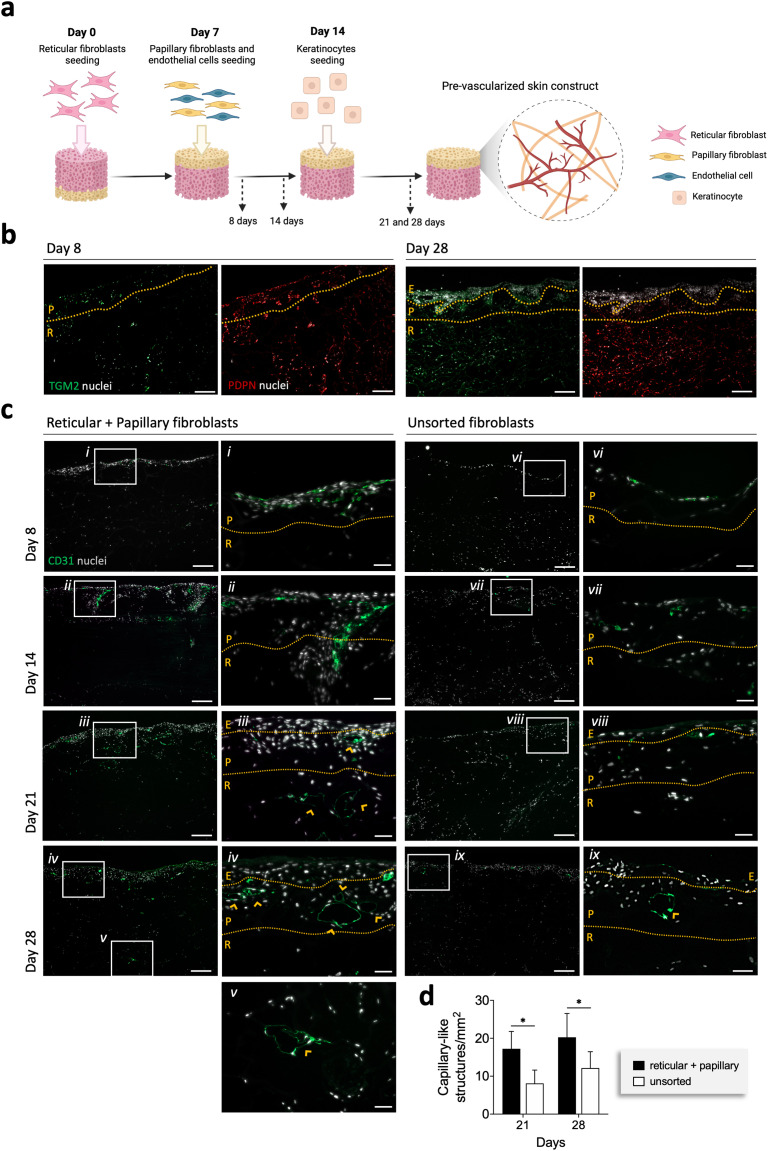
Fibroblast pre-selection fosters prevascularization of the bilayer construct. (a) Schematic representation of the reticular and papillary fibroblasts, endothelial cells and keratinocyte seeding strategy on the bilayer structure. (b) Representative immunohistochemistry images of the expression of podoplanin (PDPN) and transglutaminase-2 (TGM2) at days 8 and 28. Scale bar = 200 μm. (c) Representative immunohistochemistry images of the organization of hDMECs (CD31+, orange arrowheads) in the bilayer skin constructs prepared with reticular and papillary fibroblasts unsorted fibroblasts. (i–ix) Higher magnification of the areas outlined in the respective left panel. Nuclei were counterstained with DAPI. Scale bar = 200 μm, 50 μm. (d) Quantification of the amount of capillary-like structures at days 21 and 28. Quantitative results are expressed as the mean ± standard deviation, *p < 0.05, two-way ANOVA with Tukey multiple comparison post-test. E – epidermis, P – papillary-like dermis, R – reticular-like dermis.

**Fig. 5:**
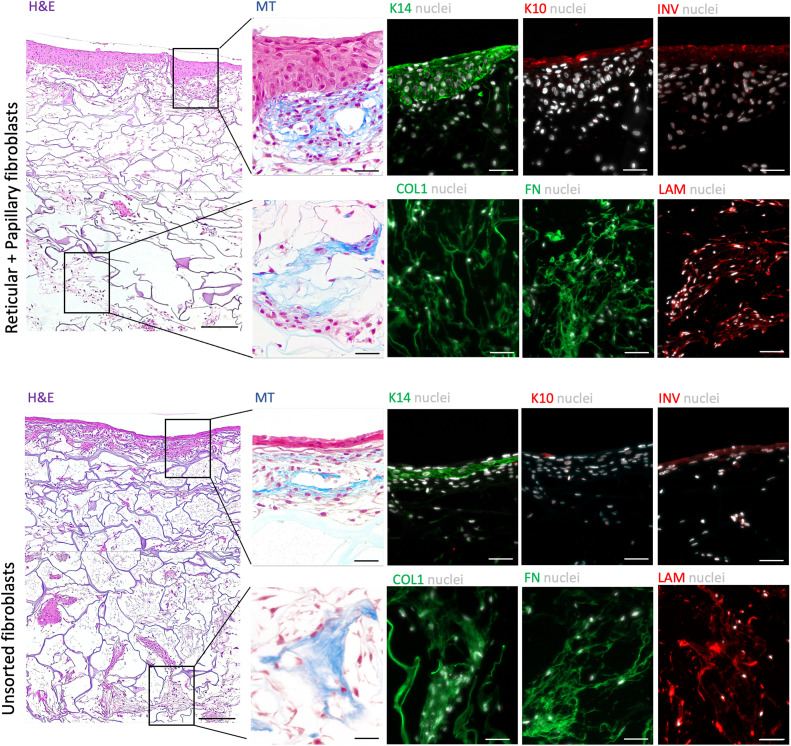
Skin-like tissue structure. Representative Haematoxylin & Eosin (H&E) images of the constructs prepared with sorted and unsorted fibroblasts, including a stratified epidermis, and an upper and lower vascularized dermis. Scale bar = 200 μm. Representative Masson's Trichrome (MT) and immunohistochemistry images of specific areas (indicated in the lower magnification H&E images) of the constructs and the expression of collagen type 1 (COL1), fibronectin (FN), laminin (LAM), cytokeratin 14 (K14), cytokeratin 10 (K10) and involucrin (INV), evidencing the stratified epidermis and the deposited extracellular matrix. Nuclei were counterstained with DAPI. Scale bar = 50 μm.

In order to validate our hypothesis, we prepared a skin-like construct in which the dermis was formed by one of two methods: (i) sorted fibroblasts and (ii) unsorted fibroblasts (Fig. 4a and ESI Fig. 8†). After the seeding of both subpopulations (day 8) fibroblasts expressing TGM2 and PDPN were found within the whole structure (Fig. 4b). Interestingly, it seems that all the cells expressing TGM2 were also positive for PDPN (Fig. 4b and ESI Fig. 9†). At day 28, however, PDPN-expressing fibroblasts (also TGM-2^+^) seemed to be mostly found in the reticular-like layer of the construct. In turn, the papillary-like part was populated by a low number of both TGM-2^+^PDPN^+^ and TGM-2^+^PDPN^−^ (Fig. 4b and ESI Fig. 9†). The hDMECs remained homogenously distributed in the upper papillary-like layer of the construct 1 day after seeding (day 8, Fig. 4b) but were invading the reticular-like layer at day 14. Organization of hDMECs into what seemed to be vessel-like structures with lumen, was observed at day 21 in the construct prepared with the sorted cell populations. These structures increased in number and remained stable throughout the culture period, while also infiltrating the reticular-like layer at day 28 (Fig. 4b). In contrast, hDMEC organization in the construct formed by the unsorted fibroblast seemed to be delayed and only at day 28 vessel-like structures with lumens could be found (Fig. 4b and c). The differences in the behavior of hDMECs in different constructs also affected keratinocyte behaviour (Fig. 5). A more differentiated epidermal layer confirmed by the expression of cytokeratin 14, cytokeratin 10 and involucrin, was observed in the construct formed by the sorted fibroblasts, in opposition to the thin layer observed in the construct containing unsorted fibroblasts. However, it seems that the ECM deposition was not affected by the pre-selection of the sub-populations of fibroblasts as shown by the deposited collagen and laminin. Overall, the organization of the skin constructs was not affected but the earlier triggering of the endothelial cells benefited the formation of a fully differentiated epidermis.

## Discussion

The papillary and reticular dermis harbours different fibroblast subpopulations responsible for establishing specific microenvironments, including distinctively vascularized dermal layers. Taking into consideration the inter-communication between fibroblast subpopulations and endothelial cells, we hypothesized that the pre-selection of the subpopulations of fibroblasts would benefit the *in vitro* generation of prevascularized skin TE constructs. We took advantage of specific markers recently identified^9^ to isolate human papillary and reticular fibroblast subpopulations with higher accuracy and reproducibility than the method used so far, based on the dermatome. These subpopulations were sorted from the lin^−^CD90^+^ population, which corresponds to around 12% of the initial fibroblast population. This low percentage of fibroblasts has already been observed by others, as the human dermis comprises endothelial, hematopoietic and neural cells and other cell types.^9^ The lin^−^CD90^+^CD39^+^CD26^−^ and lin-CD90^+^CD36^+^, respectively, the papillary and reticular fibroblasts, represented only around 4% and 1% of the initial population. Thus, the use of these cells to generate skin constructs requires their expansion to attain a higher and the relevant cell number. When we passaged the two subsets of fibroblasts, we observed the loss of key markers, as well as a decrease in their proliferative potential after a single passage, which is in agreement with other works.^9,10^ While the mechanisms associated with this phenotype switch are unknown, microenvironment alterations including pro-ECM secretion signalling have been linked to a shift in the expression of specific markers such as CD26^+^.^33,34^ In fact, potentially due to the crosstalk between the different cell types, we were able to restore the expression of TGM2 in both fibroblast subsets, while supporting their growth in 3D constructs. Still, further analyses of our constructs are needed to support this assumption. It has also been speculated that the loss of papillary-specific markers is related to the loss of synergistic Wnt-mediated crosstalk between papillary fibroblasts and keratinocytes that occurs *in vivo.*^9^ Interestingly, the ability to support the normal development of the epithelium by papillary fibroblasts after *in vitro* expansion was not affected.^14,18,35^ This is in line with our results that confirmed the formation of a more differentiated epidermis in the 3D construct formed by sorted fibroblasts, in which the papillary subpopulation was in closer contact with keratinocytes. Our work further emphasizes the importance of selecting fibroblast subsets for the appropriate development of the epidermis *in vitro*.

The dense microvasculature found in the papillary dermis is sustained by the unique microenvironment provided by papillary fibroblasts. Among other elements, the secretion of pro-angiogenic growth factors has been linked to the modulation of endothelial cell behaviour by papillary fibroblasts.^19,20^ The secretome of our papillary subpopulation was enriched in VEGF, Angio-1 and IGF-1, all angiogenic factors, which might be implicated in the observed organization of hDMECs into tubule-like structures. Indeed, both VEGF and Angio-1, known to promote endothelial cell survival, migration and organization and stabilization of capillary-like structures,^36,37^ were also identified in the secretome of dermatomed papillary fibroblasts.^19,20^ Interestingly we also found that our papillary subpopulation secretes high levels of IGF-1, which was previously shown to be responsible for stabilizing capillary-like structure formation but not eliciting endothelial cell organization.^38^ Moreover, we found that the level of IGF-1 secreted by the unsorted fibroblasts is significantly higher than that secreted by the selected subpopulations. This, together with the lower number of cells of the two sub-sets in the unsorted population might explain the reduced formation of capillary-like structures in the presence of their conditioned medium. The angiogenic secretome of dermatomed papillary fibroblasts was shown to be significantly more efficient in promoting capillary-like formation than that of the reticular fibroblasts.^19,20^ Surprisingly, we observed strong capillary-like formation elicited by the reticular subset conditioned medium, which has high levels of VEGF and HGF. While this would not be expected, other works with dermatomed reticular fibroblasts have also shown varied levels of these two growth factors,^19,20^ which, together with the different isolation procedures, hinders the comparison between conditions. Like VEGF, HGF is known to promote endothelial cell migration and organization into tubule-like structures,^39,40^ therefore, our results might be a consequence of a synergistic effect of the two.

The microenvironment generated by the fibroblast subpopulations as positive regulators of capillary-like structure formation goes beyond their paracrine effect, as their distinct ECM signature can also prompt alterations in endothelial cells.^20^ Herein we found that the ECM deposited by the papillary subset was richer in collagenous and non-collagenous proteins, which might explain the early development of vessel-like structures in our bilayer constructs generated from pre-selected fibroblasts. *In vitro*, collagens and other proteins, such as fibronectin, act synergistically to induce endothelial cell differentiation and capillary-like structure formation by interacting with specific integrins from these cells, while eliciting their adhesion, survival and migration.^41^ Moreover, the ECM produced by the reticular subset was rich in laminin. This protein is known to trigger the migration of endothelial cells through the activation of α3β1 integrin in these cells^42,43^ to expand the vessel-like structures formed.^44^ Therefore, the laminin-rich ECM might have prompted the migration of endothelial cells, initially seeded in the papillary layer, to the reticular part beneath during culture, while also playing a role in the stabilization of the formed structures^45^ at later culture times in both dermal layers. Interestingly, even though unsorted fibroblasts secreted the highest amount of collagenous proteins, their matrix was less enriched in collagen type I, which might explain the delay in the capillary-like structure formation in the model prepared with these cells. Indeed, *in vitro*, collagen I interaction with the specific α1β1 and α2β1 integrins from the endothelial cells induces precapillary cord organization and, consequently, capillary-like structure formation.^46^

Therefore, our results seem to further support the existence of an intricate signalling network responsible for the modulation of the vascular network within different dermal layers that is highly dependent on the environment generated by the respective fibroblast subpopulation.

## Conclusions

Overall, we provide evidence that pre-selection of papillary and reticular fibroblasts is relevant in promoting the *in vitro* pre-vascularization of skin TE constructs. However, the optimization of the culture conditions to allow the conservation of fibroblast subpopulation's phenotypes has not been achieved and their use in the construction of skin constructs for tissue engineering applications is hampered. Nevertheless, vascularized *in vitro* skin models built from pre-selected fibroblast subsets offer advantages in terms of representation of tissue functionality being therefore a valuable study platform.

## Author contributions

H. R. M. and A. P. M contributed to the conceptual design of the original project, designed experiments, performed analysis and validation of the data, and participated in the figure design. H. R. M. contributed to experimental execution. M. T. C contributed to isolation and characterization of the fibroblast subpopulations from skin samples provided by M. J. and R. H. L. P. d. S. and J. P. contributed to experimental preparation of the materials used in the manuscript. While the original draft preparation was done by H. R. M., review and editing were performed by A. P. M. R. L. R. and A. P. M. contributed to supervision, project administration, and funding acquisition. All authors have read and agreed to the published version of the manuscript.

## Conflicts of interest

There are no conflicts to declare.

## Supplementary Material

BM-011-D2BM02022J-s001
