# Supplementary material for: Pre-selection of fibroblast subsets prompts prevascularization of tissue engineered skin analogues
Source: Biomater Sci. 2023 May 31;11(15):5287–300. doi: 10.1039/d2bm02022j (PMC10368082; doi:10.1039/d2bm02022j)
Supplement: BM-011-D2BM02022J-s001 [file BM-011-D2BM02022J-s001.pdf]

Electronic Supplementary Material (ESI) for Biomaterials Science.
This journal is © The Royal Society of Chemistry 2023

Pre-selection of fibroblasts subsets prompt prevascularization of tissue engineered skin analogues

Helena R. Moreira[1,2], Mariana T. Cerqueira[1,2], Lucília P. da Silva[1,2], Joana Pires[1,2], Marian Jarnalo[3,4], Ricardo Horta[3,4], Rui Reis[1,2], Alexandra P. Marques[1,2,*]

[1] 3B's Research Group, I3Bs – Research Institute on Biomaterials, Biodegradables and Biomimetics, University of Minho, Headquarters of the European Institute of Excellence on Tissue Engineering and Regenerative Medicine

[2] ICVS/3B's – PT Government Associate Laboratory, Braga/Guimarães 4805-017, Portugal

[3] Department of Plastic and Reconstructive Surgery, and Burn Unity, Centro Hospitalar de São João, Porto, Portugal

[4] Faculty of Medicine - University of Porto, Portugal

* Corresponding author: apmarques@i3bs.uminho.ptapmarques@i3bs.uminho.pt

Supplementary information

Supplementary Table 1 – Fluorochrome-labeled antibodies used for cell sorting

Antibody	Brand	Ref	Host
CD324 PerCP-Cy5.5	BD Biosciences, USA	563573	Ms
CD45 PE-Cy7	BD Biosciences, USA	557748	Ms
CD31 APC-Cy7	BD Biosciences, USA	563653	Ms
CD90 FITC	eBiosciences, USA	11-0909-42	Dk
CD39 PE	BD Biosciences, USA	555464	Ms
CD26 PE-CF594	BD Biosciences, USA	565158	Ms
CD36 APC	BD Biosciences, USA	550956	Dk

Legend: Dk – donkey, Ms – mouse

Supplementary Table 2 – Fluorochrome-labeled antibodies used for flow cytometry

Antibody	Brand	Ref	Host
CD39 PE	BD Biosciences, USA	555464	Ms
CD26 PE-CF594	BD Biosciences, USA	565158	Ms
CD36 APC	BD Biosciences, USA	550956	Dk
PDNP PE	Biolegend, USA	337003	Rat
TGM2 FITC	Bioss, USA	bs-8589R-FITC	Rb

Legend: Dk – donkey, Ms – mouse, PDPN – podoplanin, Rb – rabbit, TGM2 – transglutaminase-2

Supplementary Table 3 – Immunolabelling antibodies

	Antibody	Brand	Ref	Host	Dilution
Primary antibodies	Podoplanin	Abcam, UK	ab10288	Ms	1:250
	Transglutaminase-2	Abcam, UK	ab421	Rb	1:75
	FAP	Abcam, UK	ab53066	Rb	1:100
	CD31	Dako, Denmark	M0823	Ms	1:30
	Collagen type I	Abcam, UK	ab34710	Rb	1:100
	Fibronectin	Abcam, UK	ab2413	Rb	1:100
	Laminin	Abcam, UK	ab11575	Rb	1:30
	Keratin 14	Covance, USA	PRB-160P	Rb	1:800
Secondary antibodies	Alexa fluor 488 anti-rabbit	Invitrogen, USA	A-11008	Gt	1:500
	Alexa fluor 594 anti-mouse	Invitrogen, USA	A-11032	Gt	1:500
	Alexa fluor 594 anti-rabbit	Invitrogen, USA	A-11012	Gt	1:500

Legend: FAP – fibroblast activation protein, Gt – goat, Ms – mouse, Rb – rabbit

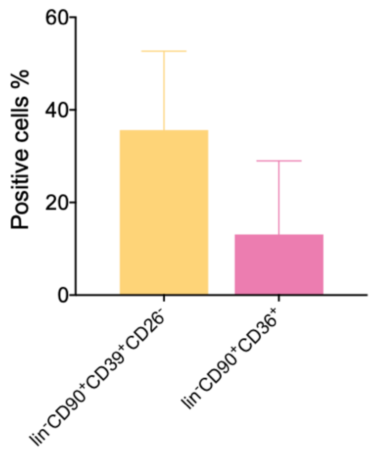

Supplementary Figure 1 – Percentage of papillary and reticular fibroblasts present in the lin$^-$CD90$^+$ fraction, N=6.

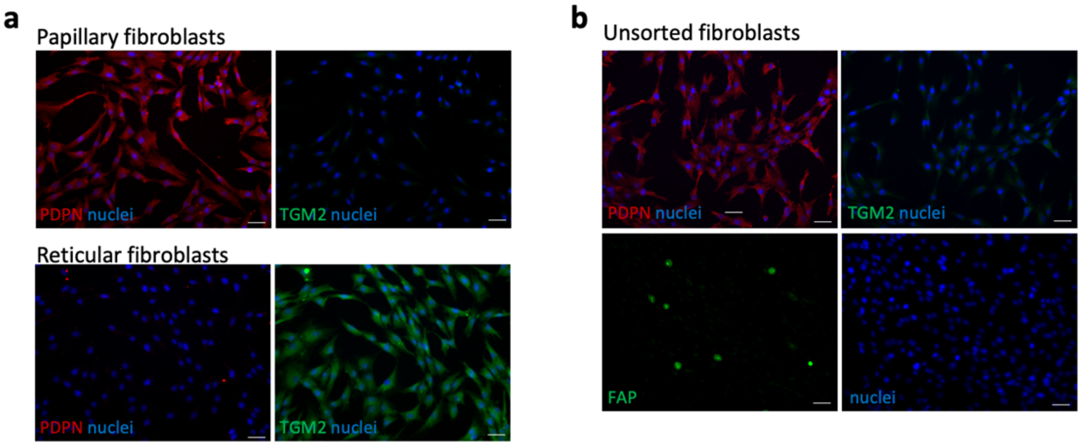

Supplementary Figure 2 – Individual immunocytochemistry images of the expression of podoplanin (PDNP), transglutaminase-2 (TGM2) and fibroblast activated protein (FAP) by **a** papillary and reticular and **b** unsorted fibroblasts populations after isolation. Scale bar = 100 µm. Nuclei were counterstained with DAPI.

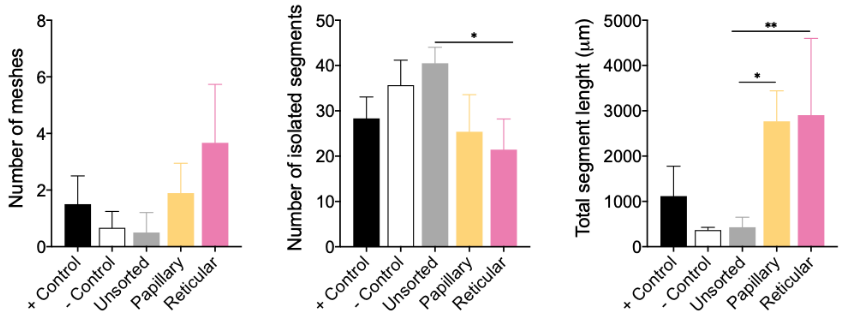

Supplementary Figure 3 – Quantification of the number of meshes, isolated segments and total segment length of the tubular-like structures formed on Matrigel by hDMECs cultured with conditioned obtained from unsorted, papillary and reticular fibroblasts after 24 h of culture. Quantitative results are expressed as the mean ± standard deviation where n = 3, * $p < 0.05$, ** $p < 0.01$, one-way ANOVA with Tukey multiple comparison post-test.

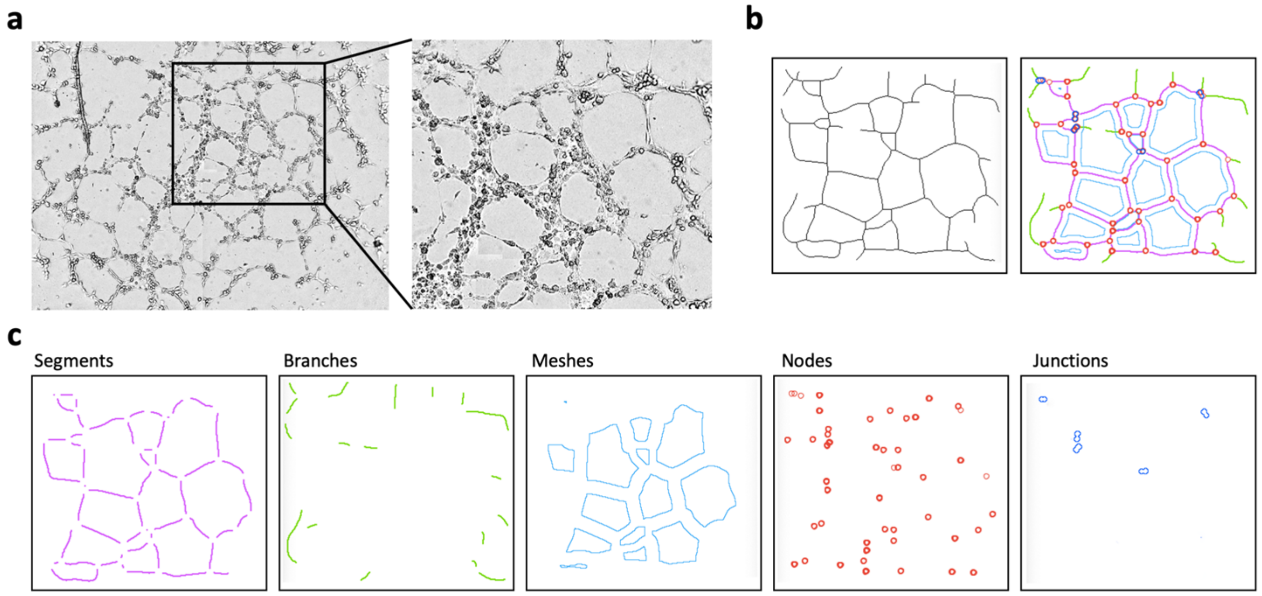

Supplementary Figure 4 – Schematic representation of the measurements obtained from the Angiogenesis Analyzer in ImageJ. a Initial image used for the measurements. b Skeleton of the image. c Detection of segments (purple) – lines connected by two nodes, branches (green) – lines connected by a node, meshes (ligh blue) – closed areas surrounded by segments, nodes (red) – minimum structure allowing a bifurcation, and junctions (dark blue) – four of more connected segments.

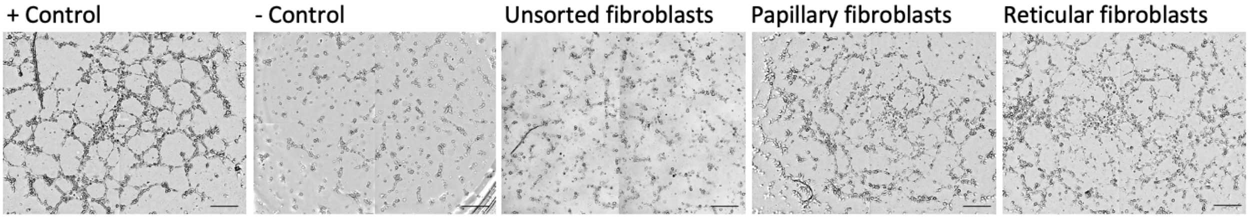

Supplementary Figure 5 – Tubular-like structures formation after 8 h by hDMECs cultured on matrigel with conditioned media obtained from unsorted, papillary and reticular fibroblasts. Controls were set with VEGF-containing medium (+ Control) and with α-MEM (- Control). Scale bar = 100 µm.

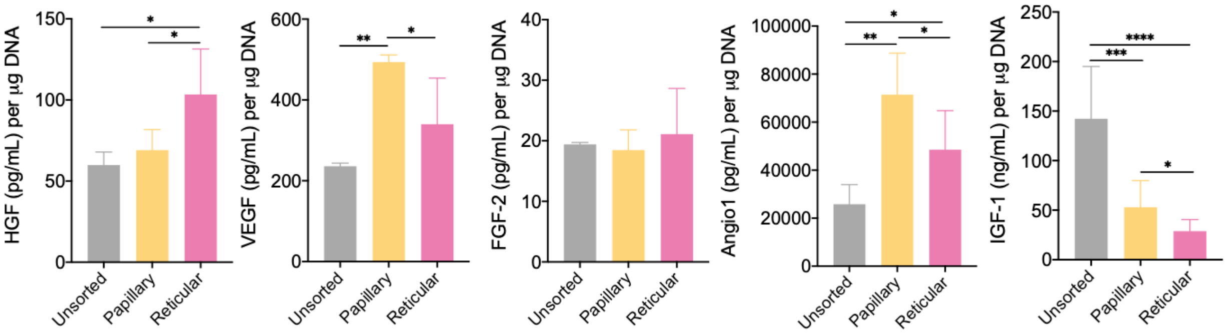

Supplementary Figure 6 – Amount of growth factors secreted by unsorted, papillary and reticular fibroblasts as quantified in the conditioned media normalized with their respective DNA amount. Quantitative results are expressed as the mean ± standard deviation where n = 3, * $p < 0.05$, ** $p < 0.01$, *** $p < 0.001$, **** $p < 0.0001$, one-way ANOVA with Tukey multiple comparison post-test.

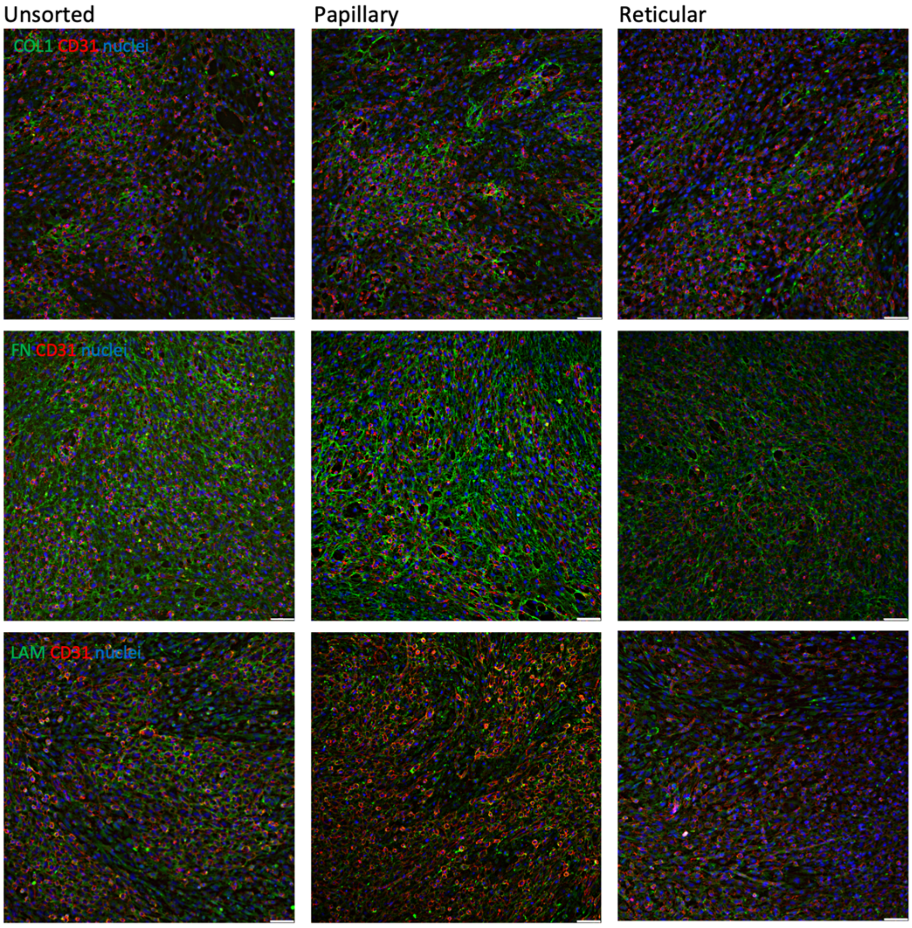

Supplementary Figure 7 – Representative merged immunocytochemistry images of the expression of collagen type I (COL1), fibronectin (FN) and laminin (LAM) by the subpopulations of fibroblasts with the organization of CD31^{+} hDMECs on the matrix of the different fibroblasts' subpopulations. Nuclei were counterstained with DAPI. Scale bar = 100 µm.

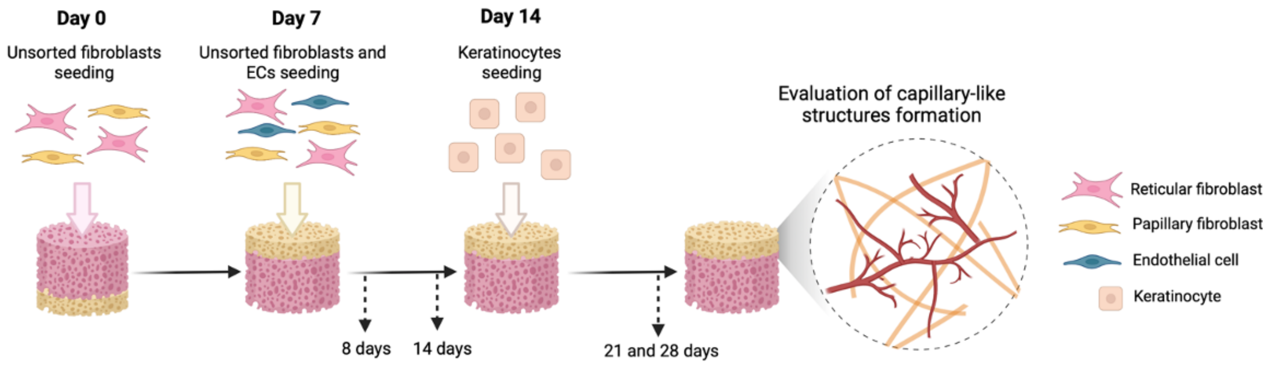

Supplementary Figure 8 – Schematic representation of the unsorted fibroblasts, endothelial cells (ECs) and keratinocytes seeding strategy on the bilayer structure.

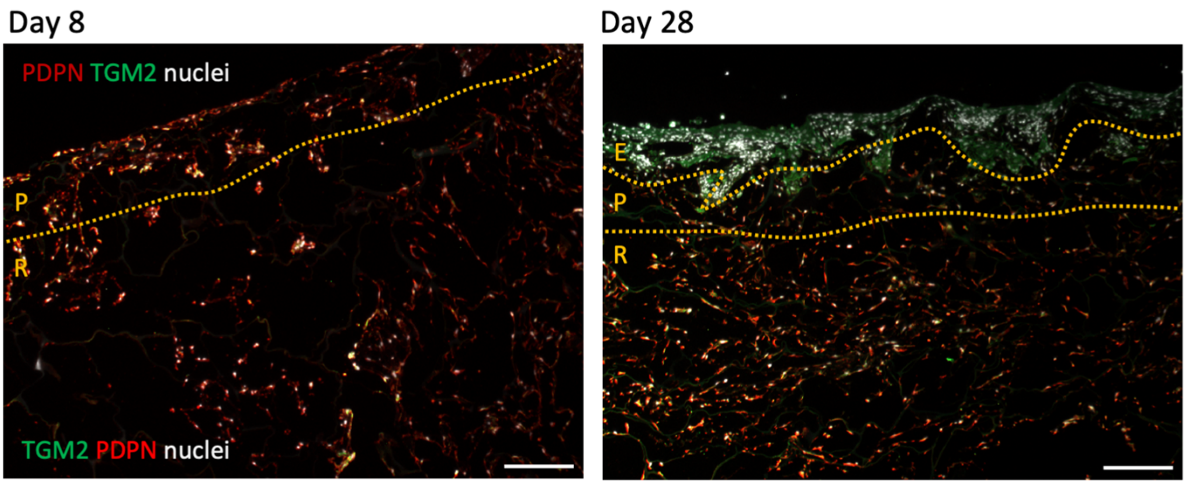

Supplementary Figure 9 – Merged immunohistochemistry images of the expression of podoplanin (PDPN) and transglutaminase-2 (TGM2) in the bilayered structures with pre-selected fibroblasts at days 8 and 28. Nuclei were counterstained with DAPI. Scale bar = 200 µm. E – epidermis, P – papillary-like dermis, R – reticular-like dermis.
